# Supplementary material for: Learning to detect AI texts and learning the limits
Source: PLoS One. 2025 Oct 15;20(10):e0333007. doi: 10.1371/journal.pone.0333007 (PMC12527182; doi:10.1371/journal.pone.0333007)
Supplement: S2 File — All data and scripts used in the study, some additional descriptive statistics, scalable formate figures. (ZIP) [file pone.0333007.s002.tgz › data and scripts/OSF/scripts/analysis/Demographics/correctness_Demographics-ai_quality.pdf]

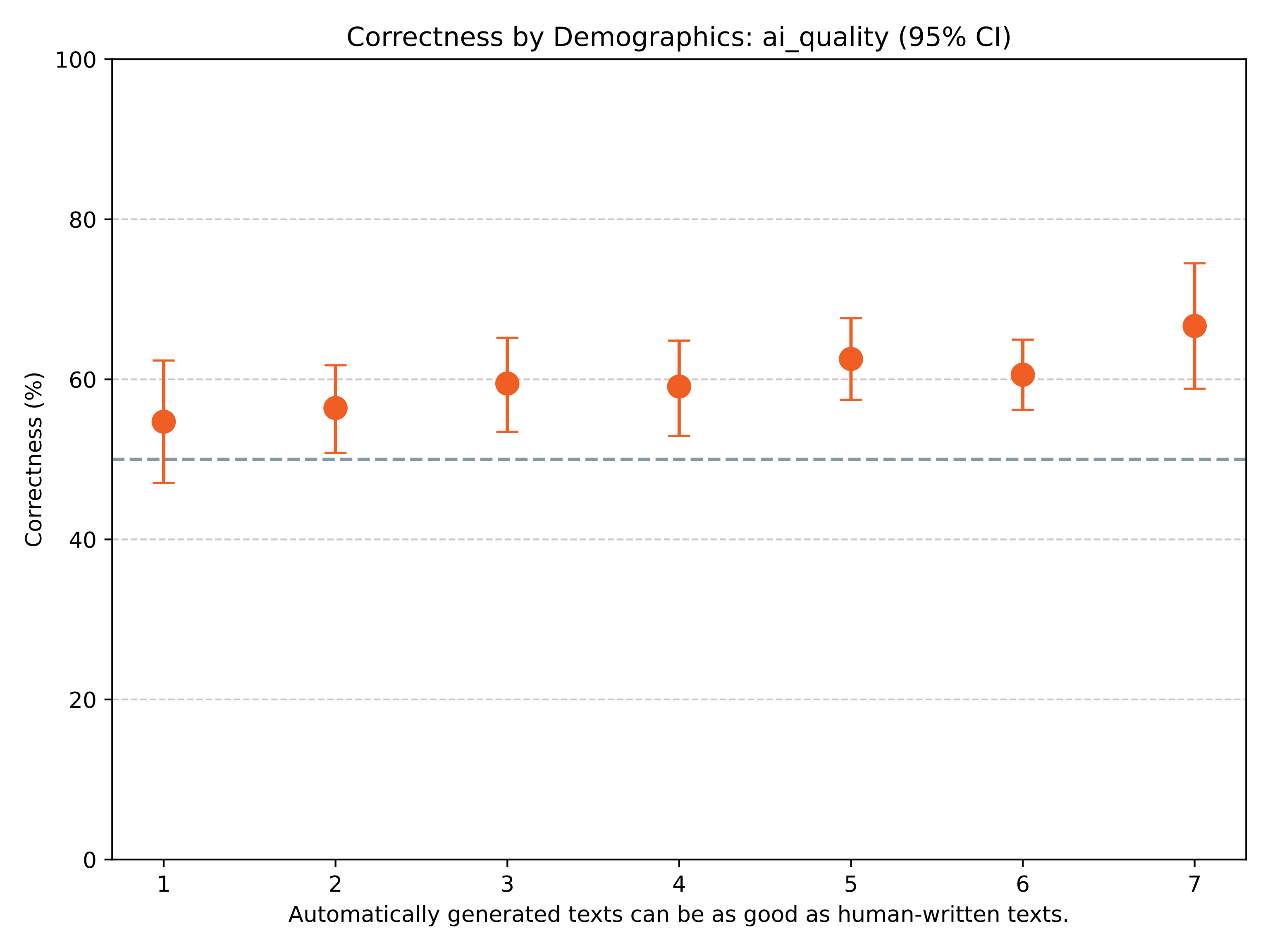
Correctness by Demographics: ai_quality (95% CI)
Correctness (%)
0
20
40
60
80
100
1
2
3
4
5
6
7
Automatically generated texts can be as good as human-written texts.
